# Prognostic impact of measurable residual clonal hematopoiesis in acute myeloid leukemia patients after allogeneic hematopoietic stem cell transplantation

**DOI:** 10.1038/s41375-023-02072-y

**Published:** 2023-10-25

**Authors:** Lara Bischof, Jule Ussmann, Juliane Grimm, Marius Bill, Dominic Brauer, Donata Backhaus, Lisa Herrmann, Maximilian Merz, Marco Herling, Klaus H. Metzeler, Georg-Nikolaus Franke, Vladan Vucinic, Uwe Platzbecker, Sebastian Schwind, Madlen Jentzsch

**Affiliations:** Department for Hematology, Cellular Therapy, Hemostaseology and Infectious Diseases, University of Leipzig Medical Center, Leipzig, Germany

**Keywords:** Translational research, Acute myeloid leukaemia

## Abstract

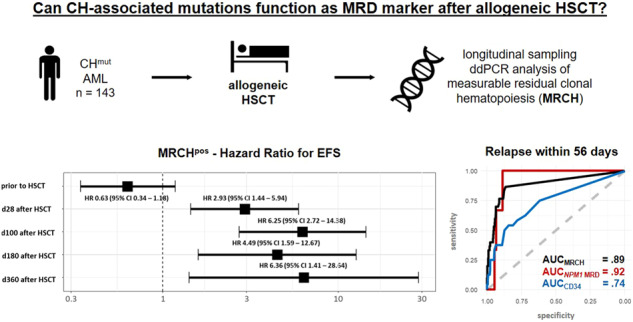

## To the Editor:

Some of the gene mutations detected during the development of myeloid neoplasm are also frequently found in elderly individuals without diagnosed hematologic malignancy and here referred to as clonal hematopoiesis (CH) of indeterminate potential (CHIP) [1]. CHIP associates with an elevated risk of developing a myeloid neoplasm [1], as compared to non-CHIP conductors [1]. Acute myeloid leukemia (AML) arises from the clonal expansion of aberrant hematopoietic stem cells, for which early events like CH-associated mutations together with later events like *FLT3*-ITD*, NRAS* or *NPM1* mutations initiate the disease. In AML, the most frequently described CH-associated mutations affect the genes *DNMT3A*, *TET2*, and *ASXL1* (‘DTA’) [2, 3]. However, also non-DTA mutations were detected in healthy individuals as well as in patients with myeloid neoplasm and described as CH, including aberrations in splicing factors like *SRSF2*, *SF3B1*, and *U2AF1*, in *JAK2*, or *IDH2* R140Q [1, 2, 4–6] At AML diagnosis, CH-associated mutations are often found at high variant allele frequencies (VAF) and frequently persist at high VAFs in remission after chemotherapy [2, 6]. Whether CH-associated mutations detected in complete remission (CR) in AML patients reflect persistent AML or are part of the physiologically aging stem cell pool is not clearly differentiated. Their persistence in CR after chemotherapy has not been linked to adverse outcomes and has very limited value for measurable residual disease (MRD) evaluation [2, 7]. However, the MRD status is an important prognostic tool when based on reliable markers, with *NPM1* mutations being the best evaluated molecular MRD marker [2, 8]. As *NPM1* mutations are only present in 30% of younger, and 20% of older AML patients [2], additional molecular MRD markers, especially in older patients, are needed for improved relapse prediction. An allogeneic hematopoietic stem cell transplantation (HSCT) results in the total replacement of a patient’s hematopoiesis. Subsequently, CH-associated mutations might function as alternative MRD markers in the post-HSCT period.

We analyzed 143 AML patients with at least one known CH-associated mutation at diagnosis receiving an allogeneic HSCT after myeloablative (mac, 13%), reduced-intensity (ric, 26%), or non-myeloablative (nma, 61%) conditioning. Median age at HSCT was 62.4 (range 31.6–76.4) years. For further patients’ characteristics see Supplementary Tables S1 and S2. Written informed consent was obtained in accordance with the Declaration of Helsinki. Median follow up after HSCT was 3.1 years.

All patients had bone marrow or peripheral blood samples for assessment of CH-associated mutations available during follow up, *i.e*. up to 28 days prior to HSCT (in CR/CRi, *n* = 101) and/or following HSCT (*n* = 88). Analyzed CH-associated mutations were in the genes *DNMT3A* (*n* = 58), *SRSF2* (*n* = 27), *IDH2* (*n* = 27)*, ASXL1* (*n* = 16), *TET2* (*n* = 17), *JAK2* (*n* = 16), *SF3B1* (*n* = 11), and *U2AF1* (*n* = 7). Mutation-specific digital droplet (dd)PCR assays were developed using a competitive probe-approach (for details see Supplementary Information and Supplementary Tables S3, S4). In patients with multiple CH-associated mutations, measurable residual clonal hematopoiesis (MRCH) positivity was defined as positivity for at least one analyzed mutation.

**Fig. 1:**
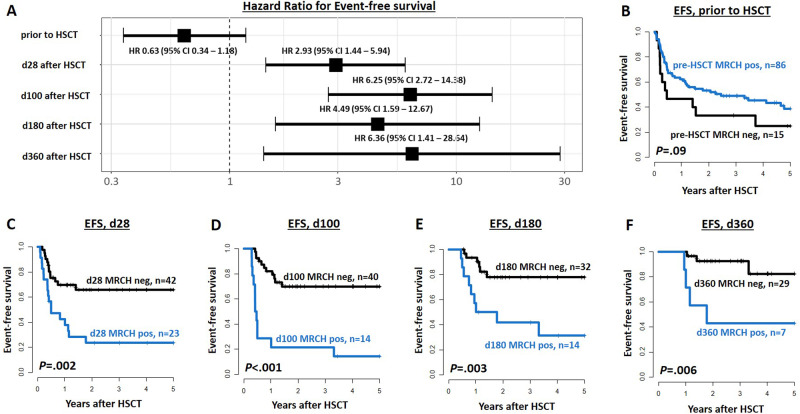
Event-free survival (EFS) of AML patients undergoing allogeneic HSCT according to the persistence of measurable residual clonal hematopoiesis (MRCH) at different time points in CR/CRi. **A** Comparison of Hazard ratios at the time points prior to HSCT, and at days 28, 100, 180, and 360 after HSCT. **B**–**F** EFS according to MRCH status at (**B**) up to 28 days prior to HSCT (*n* = 101), (**C**) 28 days after HSCT (*n* = 55), (**D**) 100 days after HSCT (*n* = 54), (**E**) 180 days after HSCT (*n* = 46), and (**F**) 360 days after HSCT (*n* = 36).

In concordance with previous studies [2, 6, 7, 9], CH-associated mutations frequently persisted in CR prior to allogeneic HSCT and did not indicate adverse outcomes (Fig. 1A, Supplementary Figs. S1A and S2A). In contrast, the majority of AML patients cleared their MRCH in CR following allogeneic HSCT. Also other reports described MRCH clearance after allogeneic HSCT in 20/21 patients [9] and *DNMT3A* mutation clearance in all patients achieving a full donor chimerism [6]. Diagnostic CH-associated mutations are usually also present at relapse as they often affect the majority of AML cells [6, 7], which was true for all patients in our study with relapse material available. In addition, relapse was preceded by at least one MRCH^pos^ sample at a median time of 53 days in 90% of relapsing patients (Supplementary Information and Supplementary Fig. S3). Interestingly, Nakamura et al. also indicated in a subset of patients (*n* = 12) an association of DTA persistence and relapse after allogeneic HSCT [10].

In cumulative outcome analyses regarding the MRCH status 28, 100, 180, and 360 days after HSCT, MRCH^pos^ status associated with a significantly higher cumulative incidence of relapse (CIR, Supplementary Fig. S1C–F) and shorter event-free survival (EFS, Fig. 1C–F) at all analyzed time points, and shorter overall survival (OS) 28 and 100 days after HSCT (Supplementary Fig. S2C–F). While relapse was frequent in MRCH^pos^ patients irrespective of the analyzed time point early after HSCT (43–65%), relapse rates in MRCH^neg^ patients continuously decreased with advancing time after HSCT (from 24% at day 28 after HSCT to 12% at day 100, 6% at day 180, and 0% at day 360, respectively, Supplementary Fig. S4).

**Fig. 2:**
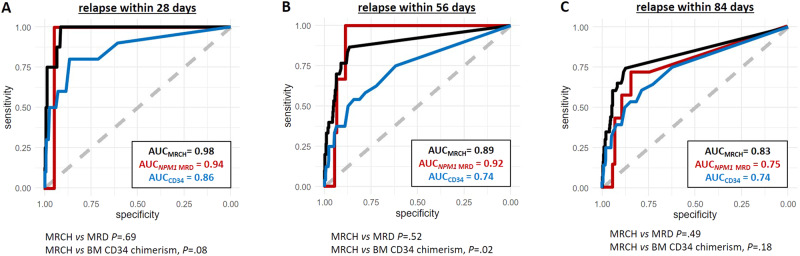
ROC curves for relapse prediction in AML patients after allogeneic HSCT according to the MRCH burden (black), *NPM1* MRD burden (red), and the bone marrow CD34 chimerism (blue) as continuous variables, measured in morphologic remission. **A** relapse within 28 days after measurement, (**B**) relapse within day 56 days after measurement, and (**C**) relapse within 84 days after measurement.

When we analyzed the relapse risk regarding the MRCH burden after HSCT, receiver operating characteristics curves showed a high efficacy of relapse prediction within 28 days (AUC_MRCH_ = 0.98), 56 days (AUC_MRCH_ = 0.89), and 84 days (AUC_MRCH_ = 0.83, Fig. 2). Of note, these predictions performed better than the bone marrow CD34 chimerism in predicting relapse within 28 and 56 days, and were not inferior to that of established *NPM1* MRD analysis (Supplementary Information and Fig. 2). For patient examples of individual MRCH and *NPM1* MRD comparisons, see Supplementary Information and Supplementary Fig. S5. A previous report has suggested *NPM1* MRD after HSCT to be the superior method compared to chimerism analysis [11], which also seems to be true for the here evaluated MRCH. Although being the best evaluated MRD marker in AML, *NPM1* mutations are lost at relapse in 10–14% of patients [8, 12]. A higher mutation stability was reported for *IDH2* und *DNMT3A* mutations (95–100%) [13], indicating that persisting CH can contribute to relapse in both *NPM1* mutated and wildtype patients [6, 12, 13]. Together with the high frequency of CH-associated mutations their use for MRD assessment can provide valuable risk evaluation in *NPM1-*negative patients and complement *NPM1* MRD in *NPM1* mutated patients after allogeneic HSCT.

The term CH-associated mutation is used inconsistently in literature. While over 30 mutated genes have been shown to behave like CHIP in healthy individuals, the DTA genes are most frequently evaluated and recognized in this context [2, 3]. To exclude distinct prognostic effects, we performed separate analyses for DTA and non-DTA MRCH. Here, we observed a similar significance for relapse prediction within 28, 56, and 84 days, as well as – although restricted by low patient numbers – in cumulative analyses at defined time points after allogeneic HSCT (Supplementary Figs. S6 and S7). One previous study analyzed DTA and non-DTA mutations (utilizing a 42 gene panel, and separating the three DTA mutations from the remaining 39 non-DTA mutations) for their value as MRD markers on days 90 and 180 post-HSCT [3]. While non-DTA MRD^pos^ was associated with a higher CIR, RFS, and OS, DTA MRD^pos^ only showed a trend towards adverse outcomes. However, sample numbers for DTA MRD^pos^ patients were low (n = 9), only analyzed at two time points after HSCT and—in contrast to our study—CH-associated mutations as well as not-CH-associated mutations were grouped together as non-DTA mutations [3]. Nevertheless, DTA MRD and non-DTA MRD VAFs were increasing similarly prior to relapse when performing monthly sample analyses [3], which matches our observations.

Finally, we analyzed the capability of relapse prediction for different CH-associated genes. Here, all genes with adequate sample numbers available (*i.e., DNMT3A*, *IDH2* R140, *SRSF2*, and *U2AF1*) were able to predict relapse within 28, 56, and 84 days. However, *SRSF2* MRCH showed a relatively low sensitivity for predicting relapse longer than 28 days after sampling (Supplementary Fig. S8). In fact, all three patients in our cohort for whom relapse was not preceded by an MRCH^pos^ sample were measured by *SRSF2* MRCH. Recently, Fabre *et al*. published data on longitudinal dynamics of CH in a cohort without hematologic malignancies. Here, *SRSF2* clones had the highest growth rate [14], matching our observations of a very short time from *SRSF2* MRCH^pos^ status to open relapse. In contrast, we observed the highest rate of MRCH^pos^ without consecutive relapse for *JAK2* mutations. *JAK2* MRCH^pos^ was often found within two months after nma or ric HSCT and turned MRCH^neg^ with reduction of immunosuppression or development of a chronic GvHD. *JAK2* mutated clones were also described to show irregular growth trajectories [14] and JAK2 activation to contribute to PD-L1 expression [15], which may explain the response of *JAK2* MRCH to graft-versus-leukemia effects and their relevance after HSCT without mac conditionings.

Our study has some limitations. While we were able to analyze MRCH mutations cumulatively, we lack sufficient patient and sample numbers to analyze all distinct genes separately. As our data indicates different dynamics and subsequent value for relapse prediction of separate genes, larger cohorts are needed to address this point. Additionally, longer follow up is needed to evaluate how sufficiently MRCH predicts late relapse after HSCT.

In conclusion, our ddPCR assays allow for replicable sensitivity with low hands-on time and costs, which is ideal for repetitive monitoring in a clinical setting. Our data suggests MRCH as a feasible marker for MRD assessment in AML patients after allogeneic HSCT, with comparable clinical value as the standard MRD marker *NPM1*. Subsequently, evaluation of MRCH may be a valuable clinical tool to detect relapse early after allogeneic HSCT, especially in patients lacking or loosing conventional MRD markers such as *NPM1*. When confirmed, MRCH can aid in guiding preemptive treatment decisions to improve outcomes after HSCT.

### Supplementary information

Supplementary Information

## Data Availability

The datasets generated during and/or analyzed during the current study are available from the corresponding author on reasonable request.
