# Supplementary material for: Prognostic impact of measurable residual clonal hematopoiesis in acute myeloid leukemia patients after allogeneic hematopoietic stem cell transplantation
Source: Leukemia. 2023 Oct 25;38(1):198–201. doi: 10.1038/s41375-023-02072-y (PMC10776391; doi:10.1038/s41375-023-02072-y)
Supplement: Supplementary file 1 — Supplementary Information [file 41375_2023_2072_MOESM1_ESM.docx]

Supplementary Information to

**Prognostic Impact of measurable residual clonal hematopoiesis in acute myeloid leukemia patients after allogeneic hematopoietic stem cell transplantation**

*Bischof et al.*

**Methods**

Patients and Treatments

Remission status at allogeneic hematopoietic stem cell transplantation (HSCT) was either first complete remission (CR) or CR with incomplete hematologic recovery (CRi, 69%), second CR/CRi (17%), or relapsed/refractory disease (14%). Genetic risk at diagnosis was assessed according to the European LeukemiaNet (ELN) 2022 classification and was 22% favorable, 26% intermediate, and 52% adverse. For further patients’ characteristics see Supplementary Table S1. MRD data analyses were approved by the Institutional Review Board of the University Hospital Leipzig (363/16-ek).

Chemotherapy protocols

The majority of AML patients received standard Cytarabine-based induction protocols, *i.e.* with conventional 7+3 alone (n=24) or in combination with Midostaurin (n=2) or Gemtuzumab ozogamicine (n=7), CPX-351^1^ (n=7) or sequential Azacytidine and OSHO induction (n=6); were treated within or according to the OSHO studies (#061 or #069,^2,3^ under or over 60 years, n=85), the Unify Trial (ClinicalTrials.gov Identifier: NCT03512197, n=2) or the Quantum first trial (n=3).^4^ Four patients received Azacytidine alone and two received Azacytidine and Venetoclax,^5^ respectively. One patient received an upfront allogeneic HSCT.

Definition of complete remission and active disease

CR was defined as the presence of <5% blasts in bone marrow, neutrophils >1.0 x 10^9^/L, platelets >100 x 10^9^/L, absence of blasts with Auer rods in peripheral blood, independence of blood transfusion and no extramedullary disease.^6^ CRi was defined as CR with platelets <100 x 10^9^/L or neutrophils <1.0 x 10^9^/L. In patients receiving allogeneic HSCT, the presence of CR or CRi was confirmed within 28 days prior to HSCT by bone marrow and peripheral blood analysis. Active disease at HSCT was defined by a persisting blast count >5% in bone marrow, persisting blasts in peripheral blood or the detection of extramedullary disease.

Allogeneic HSCT

Non-myeloablative (nma) conditioning consisted of 3x30 mg/m^2^ Fludarabine and 2 Gy total body irradiation (TBI).^7^ Myeloablative conditioning (mac) consisting of either 2x60 mg/kg body weight Cyclophosphamide and 12 Gy TBI or 5x30 mg/m^2^ Fludarabine and 8 Gy TBI. Reduced intensity conditioning (ric) consisted of either busulfan (8 mg/kg orally or 6.4 mg/kg intravenously) or Treosulfan (3x10 g/m^2^) and 5x30 mg/m^2^ Fludarabine, Fludarabine and Melphalan, Fludarabine, Thiothepa, and Melphalan, [(8)](#_CTVL001de67e159e1064dffa25f9f0c9d7f2539) or FLAMSA-based conditioning.^8–11^

Immunosuppression and graft-versus-host disease

For prevention of graft-versus-host disease (GvHD), all patients received an intravenous starting dose of 5 mg/kg body weight Cyclosporine A in two daily doses from day -1 which was adjusted to a whole-blood target level of 120-150 ng/ml for patients receiving FLAMSA conditioning or 200 ng/ml for all others.

Patients undergoing nma-HSCT additionally received Mycophenolate Mofetil 3 g per day in three daily doses in case of unrelated HSCT or 2 g per day in two daily doses in case of related HSCT. None of the patients undergoing nma-HSCT received *in vivo* T-cell depletion.

Patients receiving FLAMSA conditioning additionally received 2 g Mycophenolate Mofetil per day, which was stopped at day 28. Patients transplanted after ric and mac additionally received Methotrexate 15 mg intravenously on days +1, +3, +6, and +11 after HSCT, and ric and mac patients transplanted from an unrelated donor additionally received *in vivo* T-cell depletion with ATG 10 mg/kg per day for three days. Cyclosporine A was reduced starting on day +42 and stopped on day 120 following FLAMSA conditioning and for all others reduced starting on day +84 or day +180 following related or unrelated HSCT, respectively. After nma conditioning, Mycophenolate Mofetil was stopped at day +28 following related HSCT and tapered from days +40 to +96 following unrelated HSCT. Patients were evaluated for incidence of acute GvHD and chronic GvHD using established criteria of the Glucksberg grading system.^12^ Immunosuppression was prolonged or extended with systemic steroids in cases of GvHD (grade > 2 according to Glucksberg grading system).^12^ Requirement for acute GvHD was engraftment while requirement for chronic GvHD was engraftment and survival for at least 100 days after HSCT.

Cytogenetics, Immunophenotype and Molecular Markers at Diagnosis

Cytogenetic analyses at diagnosis were performed using standard techniques of banding and fluorescence *in situ* hybridization. Pretreatment genomic DNA was screened for the presence of *FLT3*-ITD, as well as the mutation status of the genes *CEBPA* and *NPM1* as previously described.^13,14^ In patients with adequate samples available, the diagnostic mutation status of recurrently mutated genes in myeloid malignancies was evaluated using next generation sequencing (Illumina, San Diego, CA, USA) as previously described.^13^ For patients with material available, the immunophenotype including the CD34+/CD38− cell population and *GPR56* expression at diagnosis was determined as previously described.^14,15^

MRCH and MRD assessment prior to and after allogeneic HSCT

All patients had suitable bone marrow or peripheral blood samples for assessment of clonal hematopoiesis (CH)-associated mutations available during follow up, *i.e.* up to 28 days prior to HSCT (in CR/CRi, n=101) and/or following HSCT (n=88). Median number of analyzed samples per patient after HSCT was 6 (range 1-25). At diagnosis, 111 patients had one, 27 patients had two, 4 patients had three, and 1 patient had four CH-associated mutations present, which were evaluated at and/or after HSCT.

Mutation-specific digital droplet (dd)PCR assays were developed using a competitive probe-approach for mutational hotspots (*e.g.* in *DNMT3A* R882) and patient-specific mutations for measurable residual clonal hematopoiesis (MRCH) analysis. An overview of the analyzed mutations as well as primer/probe sequences and PCR conditions is given in Supplementary Tables S3 and S4). As a result of varying assay sensitivity, MRCH positivity (MRCH^pos^) was defined as VAF ≥ 2% for *ASXL1* p.G646Wfs*12 and ≥ 0.05% for all other analyzed CH-associated mutations. In patients with multiple CH-associated mutations, MRCH^pos^ was defined as positivity for at least one analyzed mutation. For patients with a known *NPM1* mutation at diagnosis, the *NPM1* MRD was evaluated in parallel to the MRCH analyses as previously published.^16^

Evaluation of donor chimerism after allogeneic HSCT

For patients with a CD34+ AML at diagnosis, the bone marrow CD34 donor chimerism after HSCT was evaluated during the clinical routine as previously described.^17^

Statistical Analyses

Cumulative incidence of relapse (CIR) was calculated from HSCT to relapse considering its competing risk non-relapse mortality (NRM), which was calculated from HSCT to death without relapse using the Fine and Gray method.^18^ Overall survival (OS) and event-free survival (EFS) were calculated from HSCT until death from any cause and relapse or death, respectively, using the Kaplan-Meier method and groups were compared using the log-rank test. Associations with baseline clinical, demographic, and molecular features were compared using the Kruskal-Wallis test and Fisher’s exact tests for continuous and categorical variables, respectively. Receiver operating characteristic (ROC) curves were used as graphical plots to depict the predictive value of evaluated MRD, MRCH, and chimerism analysis. All statistical analyses were performed using the R statistical software platform (version 4.0.2).^19^

**Results**

CH-associated mutations in remission prior to allogeneic HSCT

In morphologic CR/CRi up to 28 days prior to HSCT the majority of patients (85%) had at least one persisting CH-associated mutation. At this time point, a MRCH^pos^ status did not associate with a higher CIR (*P*=.57, Supplementary Figure S1), shorter EFS (*P*=.09, Figure 1A), or shorter OS (*P*=.30, Supplementary Figure S2). This also remained true in separate analyses for patients with DTA and non-DTA CH-associated mutations (Supplementary Figure S6A).

Longitudinal MRCH analyses after allogeneic HSCT

Fifty-two patients (36%) relapsed during follow up after HSCT. Of those, 29 had appropriate material available at relapse. All analyzed relapse samples were positive for the diagnostic CH-associated mutations (one mutation in 25 patients, two mutations in 4 patients). Median VAF at relapse was 11.8% (range 0.18% - 68.3%).

Of all relapsing patients, 31 had at least one follow up sample in CR/CRi available prior to relapse. In 28/31 patients, impeding relapse was preceded by an MRCH^pos^ sample with a median VAF of 0.28% (range 0.06% - 12.3%) at a median of 53 days prior to relapse (Supplementary Figure S3). 3/31 patients remained MRCH^neg^ prior to relapse and had their last MRCH^neg^ samples taken 33, 35, and 132 days prior to relapse, respectively. In all three patients, MRCH was measured by a *SRSF2* mutations alone.

At least one MRCH^pos^ sample without a consecutive relapse was observed in 13/76 patients and affected the genes *JAK2* (9/13 patients), *DNMT3A* (4/18 patients), and *IDH2* (1/12 patients). Four of the nine patients with *JAK2* MRCH^pos^ samples without consecutive relapse died (5-76 days after last positive sample), one patient converted to MRCH^neg^ 5 months after stopping immunosuppression and two patients had MRCH^pos^ samples directly after ric-or nma-HSCT and converted during further follow up. All patients with *DNMT3A* MRCH^pos^ or *IDH2* MRCH^pos^ samples without consecutive relapse either developed a chronic GvHD (n=3, 30-70 days after last positive sample) or died (n=1, 104 days after last sample).

MRCH on days 28, 100, 180 and 360 after allogeneic HSCT

The prognostic relevance of MRCH was cumulatively evaluated in CR/CRi at four time points after HSCT (days 28, 100, 180, and 360). In comparison to MRCH^neg^ patients, patients with detectable MRCH had a significantly higher CIR (day 28, *P*=.002; day 100, *P*<.001; day 180, *P*=.004; and day 360 *P*<.001, Supplementary Figure S1), and shorter EFS (day 28, *P*=.002; day 100, *P*<.001; day 180, *P*=.003; and day 360 *P*=.006; Figure 1C-F). MRCH detection was significantly associated with shorter OS at day 28 and at day 100 (*P*=.006 and *P*<.001, respectively, Supplementary Figure S2), and showed a trend towards shorter OS at day 180 after HSCT (*P*=.09). Although restricted by low sample numbers, similar results were seen when we analyzed MRCH by DTA and non-DTA CH-associated mutations separately (Supplementary Figure S6).

While the overall risk of relapse remained high in MRCH^pos^ patients irrespective of the measured time point during post-HSCT follow up (43-65%), the risk of relapse in MRCH^neg^ patients continuously decreased with time after allogeneic HSCT (from 24% for MRCH^neg^ patients at day 28 after HSCT, to 12% at day 100, to 6% at day 180, to 0% at day 360, respectively, Supplementary Figure S4).

MRCH detection and clinical parameters

Comparing the clinical characteristics of patients with or without at least one MRCH^pos^ sample during the first year after allogeneic HSCT, patients with at least one MRCH^pos^ sample had a higher expression of immature antigens at diagnosis (CD34, *P*=.02; CD34+/CD38-, *P*=.07 and *GPR56*, *P*=.005), a higher serum LDH (*P*=.03) and a trend towards higher WBC (*P*=.06) at diagnosis. Prior to HSCT, they were also more likely to be transplanted without achieving a CR/CRi (*P*=.03) and by trend more likely to be *NPM1* MRD^pos^ at HSCT (*P*=.06, Supplementary Table S2). In contrast, neither the cytogenetic (*P*=.53) nor the ELN2022 risk at diagnosis (*P*=.53) were significantly different between patients with or without a MRCH^pos^ sample after allogeneic HSCT.

Relapse risk prediction comparing MRCH, *NPM1* MRD and CD34 chimerism

Using ROC curves, we compared the value of relapse prediction after HSCT within 28, 56, and 84 days by MRCH, *NPM1* MRD, and the bone marrow CD34 chimerism. Detectable MRCH (continuous values above the limit of detection) was a feasible marker for relapse prediction within 28 days (AUC_MRCH_ = 0.98), 56 days (AUC_MRCH_ = 0.89), and 84 days (AUC_MRCH_ = 0.83, Figure 2). MRCH detection was of similar value as the *NPM1* MRD status (ROC comparison: relapse within 28 days *P*=.69, relapse within 56 days *P*=.52, and relapse within 84 days *P*=.49). In comparison to the CD34 chimerism (continuous values), MRCH was significantly superior in predicting relapse within 56 days (*P*=.02), as well as within 28 days by trend (*P*=.08) and was comparable in predicting relapse within 84 days (*P*=.18). In separate analyses for DTA and non-DTA CH-associated mutations, no significant differences for relapse prediction within 28 days (AUC_DTA_ = 0.96 *vs* AUC_non-DTA_ = 0.96, *P*=.37), 56 days (AUC_DTA_ = 0.89 *vs* AUC_non-DTA_ = 0.88, *P*=.86), and 84 days (AUC_DTA_ = 0.85 *vs* AUC_non-DTA_ = 0.81, *P*=.59) were observed (Supplementary Figure S5).

For patient examples of individual comparisons between MRCH and *NPM1* MRD, please see the Supplementary Material and Supplementary Figure S7.

Relapse risk prediction for separate mutations

For genes with sufficient sample numbers, the prognostic relevance of distinct gene mutations was analyzed (i.e. *DNMT3A*, *IDH2* R140, *SRSF2*, and *U2AF1,* Supplementary Figure S8). While in general all genes were able to provide a good estimate for relapse prediction within the next 28, 56, and 84 days, *DNMT3A*, *IDH2* R140, and *U2AF1* showed high sensitivity and specificity (AUC for all time points > 0.80), while the predictive value of *SRSF2* mutations decreased with time after measurement (relapse within 28 days AUC*_SRSF2_* = 0.99, within 56 days AUC*_SRSF2_* = 0.78, and within 84 days AUC*_SRSF2_* = 0.73, mostly due to decreasing sensitivity). This may indicate that *SRSF2* MRCH conversion precedes an impeding relapse with a shorter latency time than the other analyzed genes.

Correlation of MRCH with published MRD markers

Of the patients with samples available after allogeneic HSCT, 18 had a concomitant *NPM1* mutation, which were measured for concomitant MRD follow up. Of those, eight patients relapsed: six patients showed a simultaneous *NPM1* MRD and MRCH conversion (for depicted examples, see Supplementary Figure S6), one patient had a MRCH conversion after *NPM1* MRD conversion and one patient suffered a *NPM1* MRD^neg^ relapse, which was indicated by their *IDH2* R140Q and *DNMT3A* R882H mutations 112 and 199 days prior to relapse, respectively. Of the patients in remission, one patient showed simultaneous MRCH and *NPM1* MRD conversion and one patient a *NPM1* conversion alone, but died from GvHD without relapse, while the rest continued to stay MRCH^neg^ and *NPM1* MRD^neg^.

**References**

1 Lancet JE, Uy GL, Cortes JE, Newell LF, Lin TL, Ritchie EK *et al.* Cpx-351 (cytarabine and daunorubicin) liposome for injection versus conventional cytarabine plus daunorubicin in older patients with newly diagnosed secondary acute myeloid leukemia. *J Clin Oncol* 2018; **36**: 2684–2692.

2 Büchner T, Schlenk RF, Schaich M, Doḧner K, Krahl R, Krauter J *et al.* Acute Myeloid Leukemia (AML): Different treatment strategies versus a common standard arm - Combined prospective analysis by the German AML Intergroup. *J Clin Oncol* 2012; **30**: 3604–3610.

3 Niederwieser D, Hoffmann VS, Pfirrmann M, Al-Ali HK, Schwind S, Vucinic V *et al.* Comparison of Treatment Strategies in Patients over 60 Years with AML: Final Analysis of a Prospective Randomized German AML Intergroup Study. [abstract]. In: *Blood*. 2016, p 1066.

4 Erba HP, Montesinos P, Kim H-J, Patkowska E, Vrhovac R, Žák P *et al.* Quizartinib plus chemotherapy in newly diagnosed patients with <em>FLT3</em>-internal-tandem-duplication-positive acute myeloid leukaemia (QuANTUM-First): a randomised, double-blind, placebo-controlled, phase 3 trial. *Lancet* 2023; **401**: 1571–1583.

5 DiNardo CD, Jonas BA, Pullarkat V, Thirman MJ, Garcia JS, Wei AH *et al.* Azacitidine and Venetoclax in Previously Untreated Acute Myeloid Leukemia. *N Engl J Med* 2020; **383**: 617–629.

6 Döhner H, Estey EH, Amadori S, Appelbaum FR, Büchner T, Burnett AK *et al.* Diagnosis and management of acute myeloid leukemia in adults: Recommendations from an international expert panel, on behalf of the European LeukemiaNet. *Blood* 2010; **115**: 453–474.

7 Niederwieser D, Maris M, Shizuru JA, Petersdorf E, Hegenbart U, Sandmaier BM *et al.* Low-dose total body irradiation (TBI) and fludarabine followed by hematopoietic cell transplantation (HCT) from HLA-matched or mismatched unrelated donors and postgrafting immunosuppression with cyclosporine and mycophenolate mofetil (MMF) can induce dura. *Blood* 2003; **101**: 1620–1629.

8 Kröger N, Iacobelli S, Franke GN, Platzbecker U, Uddin R, Hübel K *et al.* Dose-reduced versus standard conditioning followed by allogeneic stem-cell transplantation for patients with myelodysplastic syndrome: A prospective randomized phase III study of the EBMT (RICMAC Trial). *J Clin Oncol* 2017; **35**: 2157–2164.

9 Bryant A, Nivison-Smith I, Pillai ES, Kennedy G, Kalff A, Ritchie D *et al.* Fludarabine Melphalan reduced-intensity conditioning allotransplanation provides similar disease control in lymphoid and myeloid malignancies: Analysis of 344 patients. *Bone Marrow Transplant* 2014; **49**: 17–23.

10 Duque-Afonso J, Ihorst G, Waterhouse M, Zeiser R, Wäsch R, Bertz H *et al.* Comparison of reduced-toxicity conditioning protocols using fludarabine, melphalan combined with thiotepa or carmustine in allogeneic hematopoietic cell transplantation. *Bone Marrow Transplant* 2020; : 1–11.

11 Pfrepper C, Klink A, Behre G, Schenk T, Franke G-N, Jentzsch M *et al.* Risk factors for outcome in refractory acute myeloid leukemia patients treated with a combination of fludarabine, cytarabine, and amsacrine followed by a reduced-intensity conditioning and allogeneic stem cell transplantation. *J Cancer Res Clin Oncol* 2016; **142**: 317–324.

12 Glucksberg H, Storb R, Fefer A, Buckner CD, Neiman PE, Clift RA *et al.* Clinical manifestations of graft-versus-host disease in human recipients of marrow from HL-A-matched sibling donors. 1974; : 295–304.

13 Grimm J, Bill M, Jentzsch M, Beinicke S, Häntschel J, Goldmann K *et al.* Clinical impact of clonal hematopoiesis in acute myeloid leukemia patients receiving allogeneic transplantation. *Bone Marrow Transplant* 2019; **54**. doi:10.1038/s41409-018-0413-0.

14 Jentzsch M, Bill M, Nicolet D, Leiblein S, Schubert K, Pless M *et al.* Prognostic impact of the CD34+/CD38− cell burden in patients with acute myeloid leukemia receiving allogeneic stem cell transplantation. *Am J Hematol* 2017; **92**: 388–396.

15 Jentzsch M, Bill M, Grimm J, Schulz J, Schuhmann L, Brauer D *et al.* High expression of the stem cell marker GPR56 at diagnosis identifies acute myeloid leukemia patients at higher relapse risk after allogeneic stem cell transplantation with the CD34+/CD38- population. *Haematologica* 2020; **105**: e507.

16 Bill M, Grimm J, Jentzsch M, Kloss L, Goldmann K, Schulz J *et al.* Digital droplet PCR-based absolute quantification of pre-transplant NPM1 mutation burden predicts relapse in acute myeloid leukemia patients. *Ann Hematol* 2018; **97**: 1757–1765.

17 Hell S, Jentzsch M, Franke GN, Jäkel N, Schulze S, Edelmann J *et al.* Prospective phase II study of preemptive chimerism-driven reduction of immunosuppression after non-myeloablative conditioning—Eudract #: 2007-002420-15. *Bone Marrow Transplant* 2022; **57**: 824–826.

18 Gray RJ. A Class of K-Sample Tests for Comparing the Cumulative Incidence of a Competing Risk. *Ann Stat* 1988; **16**: 1141–1154.

19 R Development Core Team. R: A language and environment for statistical computing. Vienna, Austria. 2017. doi:R Foundation for Statistical Computing, Vienna, Austria. ISBN 3-900051-07-0, URL http://www.R-project.org.

20 Grimm J, Jentzsch M, Bill M, Backhaus D, Brauer D, Küpper J *et al.* Clinical implications of SRSF2 mutations in AML patients undergoing allogeneic stem cell transplantation. *Am J Hematol* 2021; **96**: 1287–1294.

21 Jentzsch M, Grimm J, Bill M, Küpper J, Backhaus D, Brauer D *et al.* Measurable residual disease of canonical versus non-canonical DNMT3A , TET2 , or ASXL1 mutations in AML at stem cell transplantation. *Bone Marrow Transplant* 2021; : 30–32.

22 Bill M, Jentzsch M, Grimm J, Schmalbrock LK, Küpper J, Backhaus D *et al.* Impact of IDH Mutation Detection at Diagnosis and in Remission in AML Undergoing Allogeneic Transplantation. *Blood Adv*.

**Supplementary Tables**

**Supplementary Table S1.** Patient’ characteristics.

|  | **All patients**  **n=143** |
| --- | --- |
| Age at HSCT, years  median (range) | 62.4 (31.6-76.4) |
| Sex, n (%)  male  female | 78 (55)  65 (45) |
| Disease origin, n (%)  secondary/treatment related  *de novo* | 53 (37)  90 (63) |
| Hemoglobin at diagnosis, g/dL  median (range) | 8.5 (4.5-13.4) |
| Platelet count at diagnosis, x 10^9^/L  median (range) | 72 (3-950) |
| WBC at diagnosis, x 10^9^/L  median (range) | 11.6 (0.8-385) |
| Blood blasts at diagnosis, %  median (range) | 22 (0-97) |
| Bone marrow blasts at diagnosis, %  median (range) | 50 (4.6-5) |
| CD34+/CD38- cell burden, at diagnosis, %  median (range) | 0.3 (0-89) |
| LDH at diagnosis, ukat/l  median (range) | 6.9 (1.5-37) |
| Normal karyotype, n (%)  absent  present | 62 (46)  75 (55) |
| Complex karyotype, n (%)  absent  present | 127 (94)  8 (6) |
| ELN2022 risk, n (%)  favorable  intermediate  adverse | 28 (22)  33 (26)  67 (52) |
| *ASXL1* mutation, n (%)  wild type  mutated | 78 (80)  20 (20) |
| *CEBPA* mutation, n (%)  wild type  mutated | 115 (88)  16 (12) |
| *DNMT3A* mutation, n (%)  wild type  mutated | 57 (49)  59 (51) |
| *FLT3*-ITD mutation, n (%)  wild type  mutated | 111 (79)  29 (21) |
| *FLT3*-TKD mutation, n (%)  wild type  mutated | 124 (91)  12 (9) |
| *IDH1* mutation, n (%)  wild type  mutated | 109 (87)  17 (13) |
| *IDH2* mutation, n (%)  wild type  mutated | 92 (75)  31 (25) |
| *JAK2* mutation, n (%)  wild type  mutated | 67 (80)  17 (20) |
| *NPM1* mutation, n (%)  wild type  mutated | 89 (64)  50 (36) |
| *RUNX1* mutation, n (%)  wild type  mutated | 70 (77)  21 (23) |
| *SF3B1* mutation, n (%)  wild type  mutated | 68 (86)  11 (14) |
| *SRSF2* mutation, n (%)  wild type  mutated | 74 (70)  31 (30) |
| *TET2* mutation, n (%)  wild type  mutated | 60 (71)  25 (29) |
| *TP53* mutation, n (%)  wild type  mutated | 87 (98)  2 (2) |
| *U2AF1* mutation, n (%)  wild type  mutated | 69 (91)  7 (9) |
| Remission status at HSCT, n (%)  CR/CRi 1  CR/CRi 2  PR/relapsed/refractory | 99 (69)  24 (17)  20 (14) |
| *NPM1* MRD status at HSCT, n (%)  negative  positive | 25 (58)  18 (42) |
| Conditioning regimen, n (%)  nma  ric  mac | 87 (61)  37 (26)  19 (13) |
| HCT-CI Score, n (%)  0  1/2  ≥ 3 | 50 (37)  57 (41)  29 (21) |
| Donor type, n (%)  matched related  unrelated, HLA matched  HLA mismatched  haploidentical | 16 (11)  90 (63)  32 (22)  5 (4) |
| Donor sex*,* n (%)  female into male  all others | 23 (16)  120 (84) |
| CMV status, n (%)  recipient + / donor –  all others | 48 (34)  94 (66) |
| Acute GvHD ≥ grade 2, n (%)  absent  present | 94 (73)  34 (27) |
| Chronic GvHD, n (%)  absent  limited  extended | 56 (50)  18 (16)  38 (34) |

*Abbreviations: CMV, cytomegalovirus; CR, complete remission; CRi, complete remission with incomplete peripheral recovery; ELN, European Leukemia Net; GvHD, graft-versus-host disease; Hb, hemoglobin; HLA, human leukocyte antigen; HCT-CI, hematopoietic cell transplantation comorbidity index; HSCT, hematopoietic stem cell transplantation; LDH, lactatedehydrogenase; MRD, measurable residual disease; PB, peripheral blood; PR, partial remission; WBC, white blood count.*

**Supplementary Table S2.** Clinical and genetic characteristics for patient with or without at least one sample with detectable MRCH within one year after allogeneic HSCT.

|  | **MRCH^neg^**  **n=43** | **MRCH^pos^**  **n=39** | ***P*** |
| --- | --- | --- | --- |
| Age at HSCT, years  median (range) | 58.9 (31.2-76.1) | 59.6 (33.4 – 74.0) | .66 |
| Sex, n (%)  male  female | 26 (60)  17 (40) | 16 (41)  23 (59) | .12 |
| Disease origin, n (%)  secondary/treatment related  *de novo* | 16 (37)  27 (63) | 16 (41)  23 (59) | .82 |
| Hemoglobin at diagnosis, g/dL  median (range) | 7.7 (5.6-13.4) | 8.9 (5.6-11.9) | .22 |
| Platelet count at diagnosis, x 10^9^/L  median (range) | 69 (4-488) | 69 (9-950) | .63 |
| WBC at diagnosis, x 10^9^/L  median (range) | 4.4 (0.9-385) | 15.8 (0.9-295) | .10 |
| Blood blasts at diagnosis, %  median (range) | 22 (0-97) | 41 (1-97) | .42 |
| Bone marrow blasts at diagnosis, %  median (range) | 50 (16-93) | 40 (4.6-93) | .67 |
| CD34+/CD38- cell burden at diagnosis, %  median (range) | 0.2 (0-5) | 0.9 (0-89) | .07 |
| LDH at diagnosis, ukat/l  median (range) | 6.7 (2.9-30) | 11.4 (2.2-37.2) | .03 |
| RDW at diagnosis, %  median (range) | 17.6 (13.9-30.6) | 18.2 (13.3-26.1) | .18 |
| Normal karyotype, n (%)  absent  present | 20 (48)  22 (52) | 15 (42)  21 (58) | .65 |
| Complex karyotype, n (%)  absent  present | 43 (100)  0 (0) | 33 (94)  2 (6) | .20 |
| ELN2022 risk, n (%)  favorable  intermediate  adverse | 8 (20)  9 (22)  23 (58) | 6 (21)  12 (41)  11 (38) | .20 |
| Remission status at HSCT, n (%)  CR/CRi  PR/relapsed/refractory | 38 (88)  5 (12) | 26 (67)  13 (33) | .03 |
| *NPM1* MRD status at HSCT, n (%)  negative  positive | 6 (60)  4 (40) | 1 (10)  9 (90) | .06 |
| Conditioning regimen, n (%)  nma  ric  mac | 12 (28)  20 (47)  11 (25) | 17 (44)  14 (36)  8 (20) | .35 |
| Acute GvHD ≥ grade 2, n (%)  absent  present | 31 (79)  8 (21) | 25 (74)  9 (26) | .59 |
| Chronic GvHD, n (%)  absent  limited  extended | 26 (72)  4 (11)  6 (17) | 18 (56)  4 (13)  10 (31) | .32 |

*Abbreviations: BM, bone marrow; CD, cluster of differentiation*

**Supplementary Table S3: ddPCR PCR conditions for MRCH mutations.** For every gene’s (mutation and wild type) copy number determination, the droplets were generated using the Automated Droplet Generator (BioRad). PCR was performed using the Thermocycler T100 (BioRad). The PCR consists of an initial denaturation (95°C 10 minutes) followed by 40 cycles (60 cycles or *ASXL1* G464Wfs*12) (denaturation 94°C 30 seconds; annealing/extension, please refer to the table for the used duration and temperature) with a ramp rate set to 2°C/min and a final extension (98°C 10 min).

| **Gene** | **Mutation** | **Annealing/extension** | |
| --- | --- | --- | --- |
|  |  | **Temperature** | **Time** |
| ***IDH2*** | R140Q | 49° | 2 min |
| ***SRSF2*** | P95H, L, R | as published before^20^ | |
| ***DNMT3A*** | D531G  E817*  W305*  G543D  G707D  R771fs  P743Afs*6  R726H | 46°  50.3°  50.3°  50.3°  50.3°  50.3°  51°  57° | 1 min |
|  | R882H, C | as published before^21^ | |
| ***JAK2*** | V617F | Biorad commercial assay #10042964 | |
| ***SF3B1*** | K700E | 50° cDNA  54° gDNA | 1 min |
|  | K666N, T | 51° |  |
| ***TET2*** | C1272Wfs*29  L1329R  L1872R  Q706*  L1872R  Q884*  Q962*  T970I  Y1245*  C1263R  R1452*  R1460L | 50°  50°  50°  51°  51°  52.3°  52.3°  52.3°  52.3°  52.3°  55°  55° | 1 min |
| ***U2AF1*** | S34F  Q157P | 51° | 1 min |
| ***ASXL1*** | G646Wfs*12 | as published before^21^ | |
|  | E1102D  E657*  R693*  Q706*  I617* | 50.3°  50.3°  50.3°  50.3°  51° | 1 min |

**Supplementary Table S4. Primer and probe sequences for MRCH evaluation by digital droplet PCR.**

| **mutation** | **probe sequence** | **primer forward sequence** | **primer reverse sequence** |
| --- | --- | --- | --- |
| ***ASXL1*** |  | | |
| **I617*** | **wildtype**  5’CTCGCAGACATTAAAGC3’  **mutation**  5´CTCGCAGACTAAATTAAA3´ | **cDNA and gDNA**  5´GATCATCCCCACCACGGAG3´ | **cDNA and gDNA**  5´TCGGACCTGCAGAGCAC3´ |
| **Gly646Trpfs*12** | **as published before**^21^ | | |
| **E657*** | **wildtype**  5’CCACCGATGAGGGAGGT3‘  **mutation**  5´CCACCGATTAGGGAGGT3´ | **cDNA and gDNA**  5´GAGGTCACCACTGCCATAGA3´ | **cDNA and gDNA**  5´CCTCACCACCATCACCACT3´ |
| **R693*** | **wildtype**  5’CTACAGCGAACACAACTACT3‘  **mutation**  5´CTACAGTGAACACAACTACT3´ | **cDNA and gDNA**  5´ACCCCTGGAAAGTGTACGTC3´ | **cDNA and gDNA**  5´CTACCCATTTAGAGGATAAGGC3´ |
| **Q760*** | **wildtype**  5’CAGCCATGCCAGGCCTT3‘  **mutation**  5´CAGCCATGCTAGGCCTT3´ | **cDNA and gDNA**  5´ACAGATGGGCTAGGAGATGC3´ | **cDNA and gDNA**  5´CTGAGGTTTGGGAGGACAGT3´ |
| **E1102D** | **wildtype**  5’CCTCAGTGGAGGCCACT3‘  **mutation**  5´CCTCAGTGGATGCCACT3´ | **cDNA and gDNA**  5´GTGTGCCTGTCCATGCCT3´ | **cDNA and gDNA**  5´CAACTGCATCACAAGTGGGT3´ |
| ***DNMT3A*** |  |  |  |
| **W305*** | **wildtype**  5’CTGGCCACCAGGAGAA3‘  **mutation**  5´CTGGCCATCAGGAGAA3´ | **cDNA and gDNA**  5´ATCCACCAAGACACAATGCG3´ | **cDNA and gDNA**  5´CTTTGGCATTGGGGAGCTG3´ |
| **D531G** | **wildtype**  5‘GTAGCCGTCGTCGTCGT3‘ | **gDNA**  5´TTGTTTCCGCACATGAGCAC3´ | **gDNA** 5´CTTCCCGCTGCTGTCTAGAA3´ |
|  | **mutation**  5‘GTAGCCGCCGTCGTCGT3‘ | **cDNA**  5´TTGTTTCCGCACATGAGCAC3´ | **cDNA** 5´ACATCTGCATCTCCTGTGGG3´ |
| **G543D** | **wildtype**  5’GTGGGGGCCGTGAGGT3‘  **mutation**  5´GTGGGGACCGTGAGGT3´ | **cDNA and gDNA**  5´GGAGTGTGCGTACCAGTAC3´ | **cDNA and gDNA**  5´ACCTGCAGCAGTTGTTGTTT3´ |
| **D707D** | **Wildtype**  5’GATTGGGGGCAGTCCCT3‘ | **gDNA** 5´ACGTTGCCTTTATCCTCCCA3´ | **gDNA**  5´CTTGCGAGCAGGGTTGAC3´ |
|  | **mutation**  5´GATTGGGGACAGTCCCT3´ | **cDNA**  5´AGATCATGTACGTCGGGGAC3´ | **cDNA**  5´CTTGCGAGCAGGGTTGAC3´ |
| **R736H** | **wildtype**  5‘AGTTCTACCGCCTCCTGCATG3‘  **mutation**  5´AGTTCTACCACCTCCTGAATG3´ | **gDNA**  5’GGCTTTCTCTTCCGACCTCT3‘  **cDNA**  5´CAGTCCCTGCAATGACCTCT3´ | **cDNA and gDNA**  5´CAGAAGAAGGGGCGATCAT3´ |
| **P743Afs*6** | **wildtype**  5’ATGCGCGGCCCAAGG3‘  **mutation**  5´ATGCGCGGGCCCAAG3´ | **gDNA**  5’GGCTTTCTCTTCCGACCTCT3‘  **cDNA**  5´CAGTCCCTGCAATGACCTCT3´ | **cDNA and gDNA**  5´CAGAAGAAGGGGCGATCAT3´ |
| **R771fs** | **wildtype**  5‘GACATCTCGCGATTTCTCGA 3‘  **mutation**  5´GACATCTCGATTTCTCGA3´ | **cDNA and gDNA**  5´CCATGGGCGTTAGTGACAAG3´ | **cDNA and gDNA**  5´CCATTAGTGAGCTGGCCAAA3´ |
| **E817*** | **wildtype**  5‘GCTGCAGGAGTGTCTGGA3‘  **mutation**  5´GCTGCAGTAGTGTCTGGA3´ | **cDNA**  5´TGGCATCCACTGTGAATGAT3´ | **cDNA**  5´AAATGCTGGTCTTTGCCCTG3´ |
| **R882** | **as** **published** **before**^21^ | | |
| ***IDH2*** |  | | |
| **R140Q** | **as published before**^22^ | | |
| ***JAK2*** |  | | |
| **V617F** | **was purchased from Biorad #10042964** | | |
| ***SF3B1*** |  |  |  |
| **K666N, T** | **wildtype**  5’CACACTGGTATTAAGATTGTACA3‘  **mutation c.1998G>C**  5´CACACTGGTATTAACATTGTACA3´  **mutation c.1998G>T**  5’CACACTGGTATTAATATTGTACA3‘  **mutation c.1997A>C**  5’CACACTGGTATTACGATTGTACA3‘ | **cDNA and gDNA**  5´AGCTGTGTGCAAAAGCAAGA3´ | **cDNA and gDNA**  5´AGATGGCACAGCCCATAAGA3´ |
| **K700E** | **wildtype**  5´ CAGCAGAAAGTTCGGACCA3´ | **gDNA** 5´TTGGGGCATAGTTAAAACCTG3´ | **gDNA** 5´GAGTTGCTGCTTCAGCCAAG3´ |
|  | **mutation**  5´CAGCAGGAAGTTCGGACCA3´ | **cDNA**  5´TGAACATGGTCTTGTGGATGA3´ | **cDNA**  5´AGCCAAACCCTTTCCTCTGT3´ |
| ***SRSF2*** |  | | |
| **P95H, L, R** | **as published before**^20^ | | |
| ***TET2*** |  |  |  |
| **Q706*** | **wild type**  5‘GAATCAACAGGCTTCAGAGA3´  **mutation**  5‘GAATCAATAGGCTTCAGAGA3‘ | **cDNA and gDNA**  5´GTCCCCAGTGTTGAAACAGC3` | **cDNA and gDNA**  5´AGGCTTATGTTGCAAAAGGTGT3´ |
| **Y867H** | **wild type**  5´TCACATGCAATATTTTCCAAAT3´  **mutation**  5´TCACATGCAATATTTTCCAAAT3´ | **cDNA and gDNA**  5´GCAGGAAACAAGACCCAAAA3´ | **cDNA and gDNA**  5´TGTTCTTGAAAGCACCTGTGA3´ |
| **Q884*** | **wild type**  5’TGCTTTCAAGAACAGGAGCA3‘  **mutation**  5´TGCTTTTAAGAACAGGAGCA3´ | **cDNA and gDNA**  5´AATGTGATCCCAAAGCAAGA3´ | **cDNA and gDNA**  5´CCCTGTAGAACTGAAGCTTGTTG3´ |
| **Q962*** | **wild type**  5´CAGAAGCAAGAACAGCAGCA3’  **mutation**  5´CAGAAGCAAGAATAGCAGCA3´ | **cDNA and gDNA**  5´CAAAAGCATGCTGCTCTAAGG3´ | **cDNA and gDNA**  5´ACTCAGTTTGGGGTTGCTGT3´ |
| **T970I** | **wild type**  5´CCCAAACTGAGTCTTGCCAT3’  **mutation**  5´CCCAAATTGAGTCTTGCCAT3´ | **cDNA and gDNA**  5´AGCAAGAACAGCAGCAAACA3´ | **cDNA and gDNA**  5´TTAATTGGCCTGTGCATCTG3´ |
| **Y1245*** | **wild type**  5‘GCTGACAAACTCTACTCGGAGC3’  **mutation**  5´GCTGACAAACTCTAGTCGGAGC3´ | **cDNA and gDNA**  5´TGATTCTCATCCTGGTGTGG3´ | **cDNA and gDNA**  5´ATTTCCTCAGCGTCTCGGTA3´ |
| **C1263R** | **wild type**  5‘CAATCGCCGGTGTGCC3’  **mutation**  5´CAATCGCCGGCGTGCC3´ | **gDNA**  5´GAGACGCTGAGGAATACGG3´ | **gDNA**  5´GCCCTGGGCTTCACTTACTC3´ |
| **L1329R** | **wild type**  5´TCATTTGCAAAACCTGTCCA3´ | **gDNA**  5´CACTTTATACAGGAAGAGAAACTGGAG3´ | **gDNA**  5´TGCATCAGGTGCAAGTTTCTTA3´ |
|  | **mutation**  5´TCATTTGCAAAACCTGTCCA3´ | **cDNA**  5´GTTTGCCAGAAGCAAGATCC3´ | **cDNA** 5´TGCATCAGGTGCAAGTTTCTTA3´ |
| **R1452*** | **wild type**  5’GTTCTTTTCGGCGAAAAGTCA3‘  **mutation**  5´GTTCTTTTCGGTGAAAAGTCA3´ | **cDNA and gDNA**  5´TGGAAGCTCAGGAGGAGAAA3´ | **cDNA and gDNA**  5´TTTCCTTTGTCGGCAAGTCT3´ |
| **P1460L** | **wild type**  5‘GATGTTAGCAGAGCCAGTCA3’  **mutation**  5´GATGTTAGCAGAGCTAGTCA3´ | **cDNA and gDNA**  5´TGGAAGCTCAGGAGGAGAAA3´ | **cDNA and gDNA**  5´TTTCCTTTGTCGGCAAGTCT3´ |
| **Q1547*** | **wild type**  5´CAGCAGCAGCAGCCA3´  **mutation**  5´CAGCAGTAGCAGCCA3’ | **cDNA and gDNA**  5´TACAGAAGCAGCCACCACAG3´ | **cDNA and gDNA**  5´GGTGGATCCAGAAGCAGAAT3´ |
| **L1872R** | **wild type**  5‘TCAATTCTCATTGAGTGTGC3’  **mutation**  5´TCAATTCGCACTGAGTGTGC3´ | **cDNA and gDNA**  5´GGCCGTGGCTCCAACTCAT3´ | **cDNA and gDNA**  5´TGGGATTCTTTAAAGGGGTTG3´ |
| **I1873T** | **wild type**  5´TCAATTCTCATTGAGTGTGC3’  **mutation**  5´TCAATTCTCACTGAGTGTGC3´ | **cDNA and gDNA**  5´GGCCGTGGCTCCAACTCAT3´ | **cDNA and gDNA**  5´TGGGATTCTTTAAAGGGGTTG3´ |
| ***U2AF1*** |  |  |  |
| **S34F** | **wildtype**  5’GGTGCTCTCGGTTGCA3‘  **mutation**  5´GGTGCTTTCGGTTGCA3´ | **cDNA** 5´TCAAAATTGGAGCATGTCGTCA3´ | **cDNA**  5´GTCAGCAGACTGGGAAGAGT3´ |
|  |  | **gDNA** 5´TCAAAATTGGAGCATGTCGTCA3´ | **gDNA**  5´ACAAACCTGGCTAAACGTCG3´ |
| **Q157P** | **wildtype**  5’CTGCCGTCAGTATGAGATG3‘  **mutation**  5´CTGCCGTCCGTATGAGATG3´ | **cDNA**  5´GACGGACTTCAGAGAAGCCT3´ | **cDNA**  5´AGCTCTCTGGAAATCGGCTT3´ |
|  |  | **gDNA**  5´GACGGACTTCAGAGAAGCCT3´ | **gDNA**  5´CTGTGCTCAGTCACGTCACT3´ |

*Abbreviations: cDNA, complementary DNA; gDNA, genomic DNA.*

**Supplementary Figures**

**Supplementary Figure S1.**

**
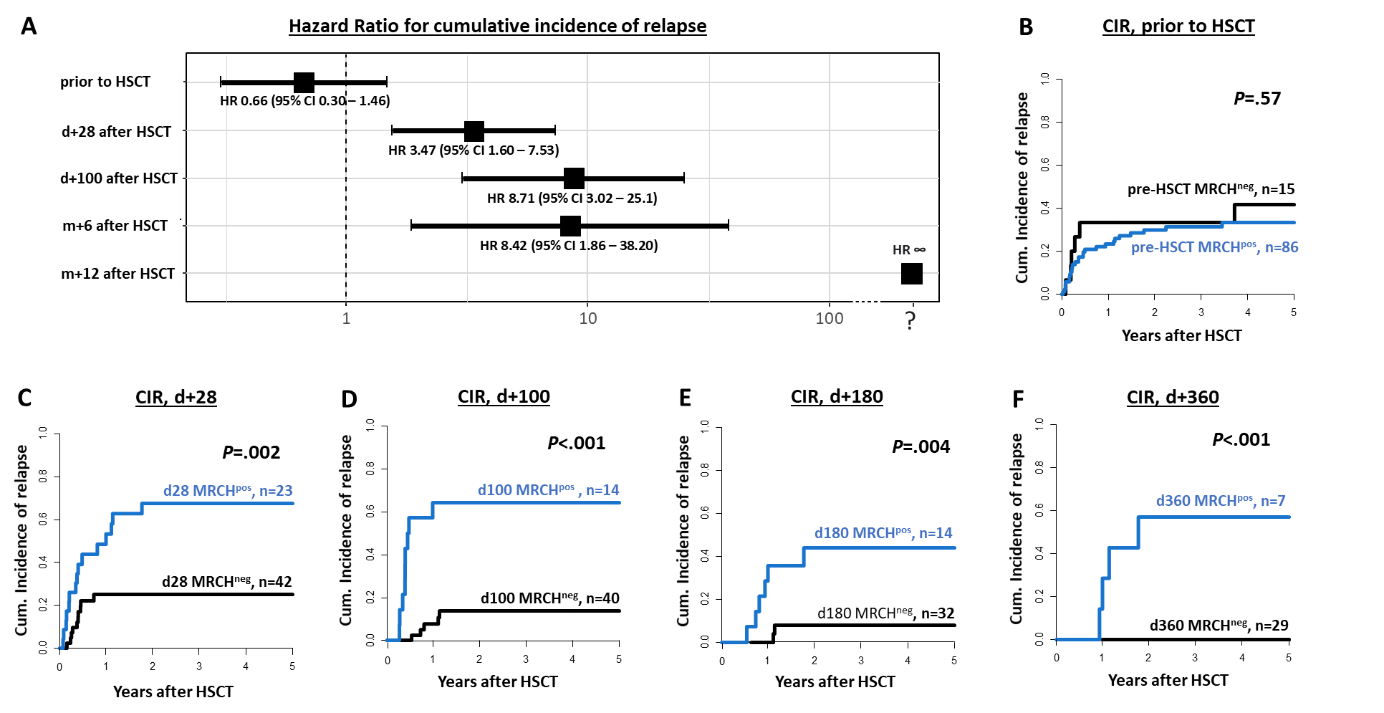
**

**Supplementary Figure S1. Cumulative incidence of relapse of AML patients undergoing allogeneic HSCT according to the persistence of measurable residual clonal hematopoiesis (MRCH) at different time points in CR/CRi. (A)** Comparison of Hazard ratios at the time points prior to HSCT, at day 28 after HSCT, at day 100 after HSCT, at day 180 after HSCT, and at day 360 after HSCT. **(B-F) Cumulative incidence of relapse at (B)** up to 28 days prior to HSCT (n=101), **(C)** 28 days after HSCT (n=55)**, (D)** 100 days after HSCT (n=54), **(E)** 180 days after HSCT (n=46), and **(F)** 360 days after HSCT (n=36).

**Supplementary Figure S2.**

**
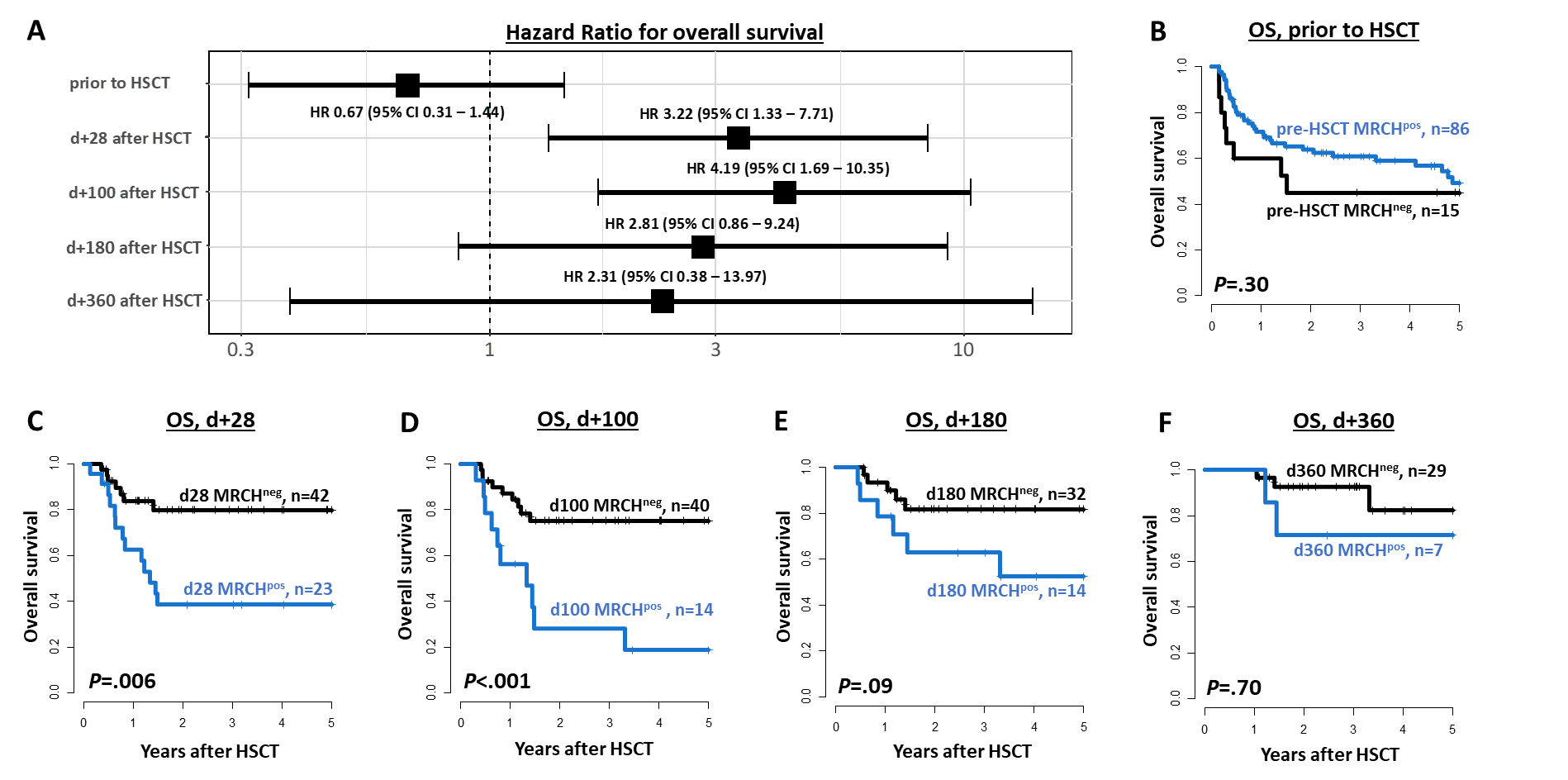
**

**Supplementary Figure S2. Overall survival in AML patients undergoing allogeneic HSCT according to the persistence of measurable residual clonal hematopoiesis (MRCH) at different time points in morphologic remission. (A)** Comparison of Hazard ratios at the time points prior to HSCT, at day 28 after HSCT, at day 100 after HSCT, at day 180 after HSCT, and at day 360 after HSCT. **(B-F) Overall survival at (B)** up to 28 days prior to HSCT (n=101), **(C)** 28 days after HSCT (n=55)**, (D)** 100 days after HSCT (n=54), **(E)** 180 days after HSCT (n=46), and **(F)** 360 days after HSCT (n=36).

**Supplementary Figure S3.**

**Supplementary Figure S3. Longitudinal assessment of MRCH during the first year after HSCT. (A)** in patients remaining in remission and **(B)** in patients relapsing after allogeneic HSCT.

**Supplementary Figure S4.**

**
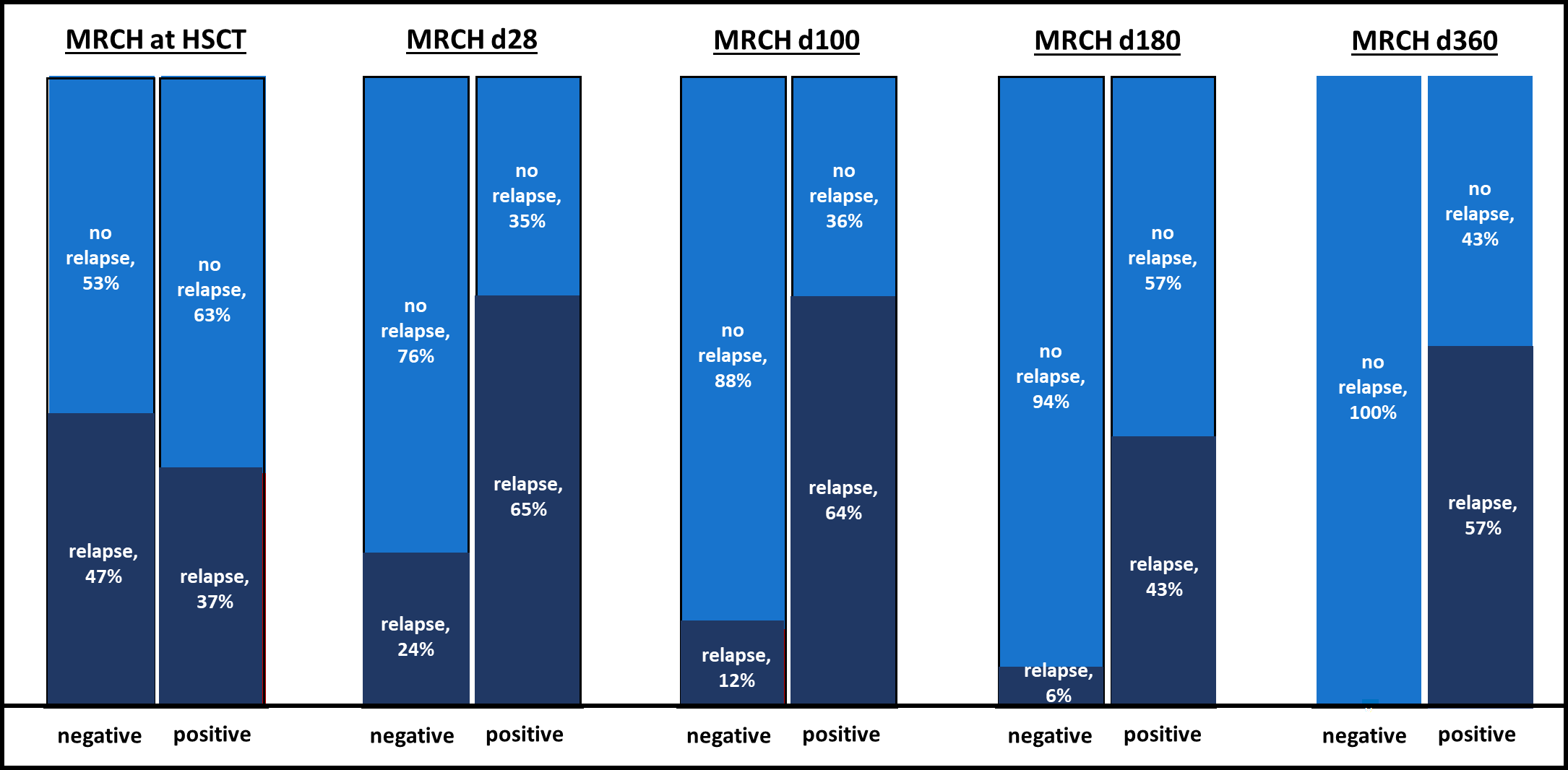
**

**Supplementary Figure S4. Risk of relapse of AML patients undergoing allogeneic HSCT according to the persistence of MRCH at HSCT, as well as 28, 100, 180, and 360 days after HSCT in CR/CRi. (dark blue)** relapse, **(blue)** no relapse

**Supplementary Figure S5.**

**
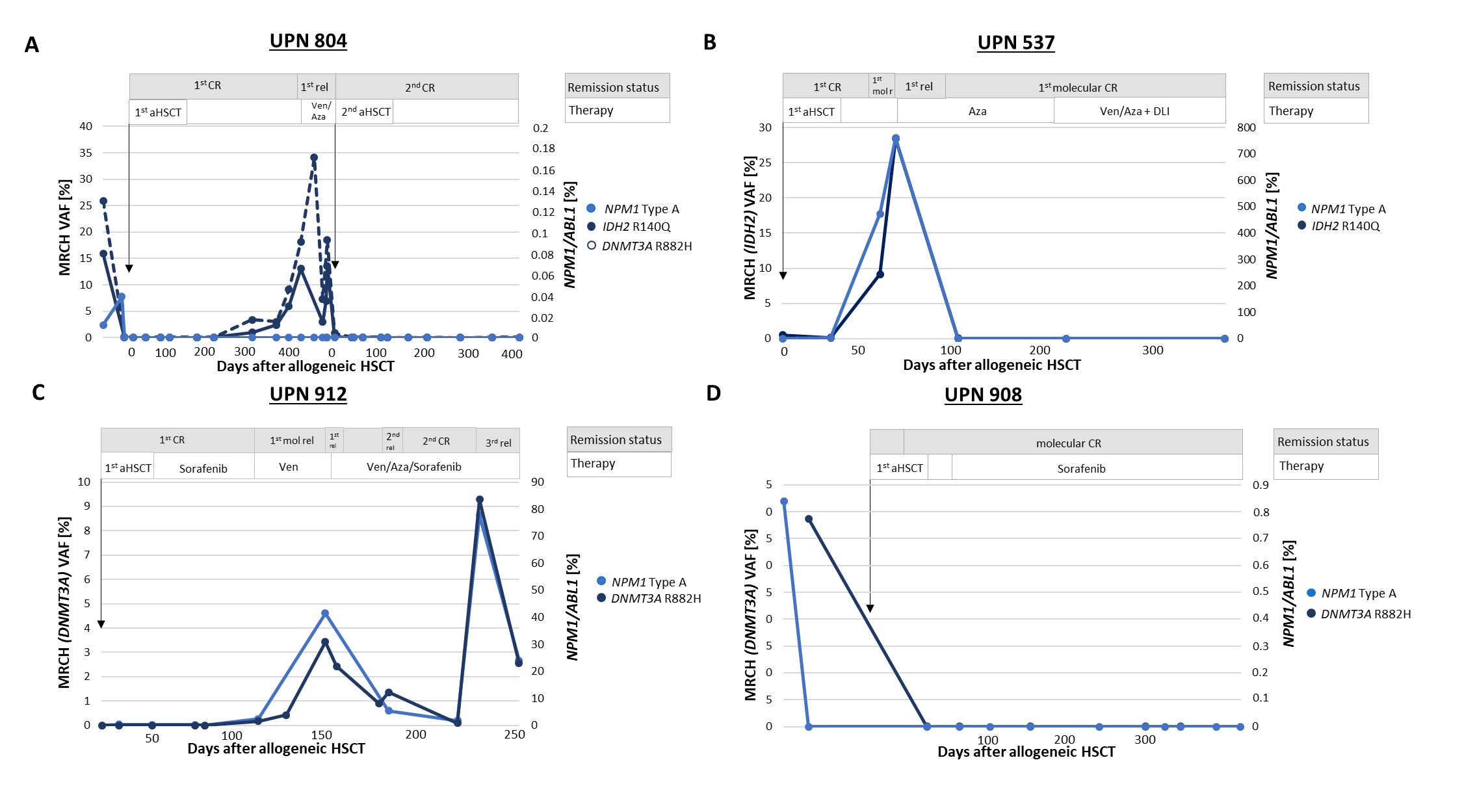
**

*Abbreviations: CR, Complete Remission; rel, relapse; aHSCT, allogeneic HSCT; mol, molecular; Venetoclax, Ven; Aza, Azacytidine; DLI; donor lymphocyte infusion*

**Supplementary Figure S5. Longitudinal dynamics of AML patients after allogeneic HSCT of MRCH or *NPM1*-mutation based MRD displayed as Variant allele frequency (VAF) and *NPM1*/*ABL1*[%], respectively. (A) UPN 804,** this patient lost their *NPM1* mutation at relapse**.** *IDH2* VAF (dark blue straight), *DNMT3A* VAF (dark blue dotted), *NPM1/ABL1* (blue), **(B) UPN 537,** *IDH2* VAF (dark blue), *NPM1/ABL1%* (blue), **(C) UPN 912**, *DNMT3A* VAF (dark blue), *NPM1/ABL1%* (blue), **(D)** **UPN 908,** *DNMT3A VAF* (dark blue), *NPM1/ABL1%* (blue).

**Supplementary Figure S6.**

**
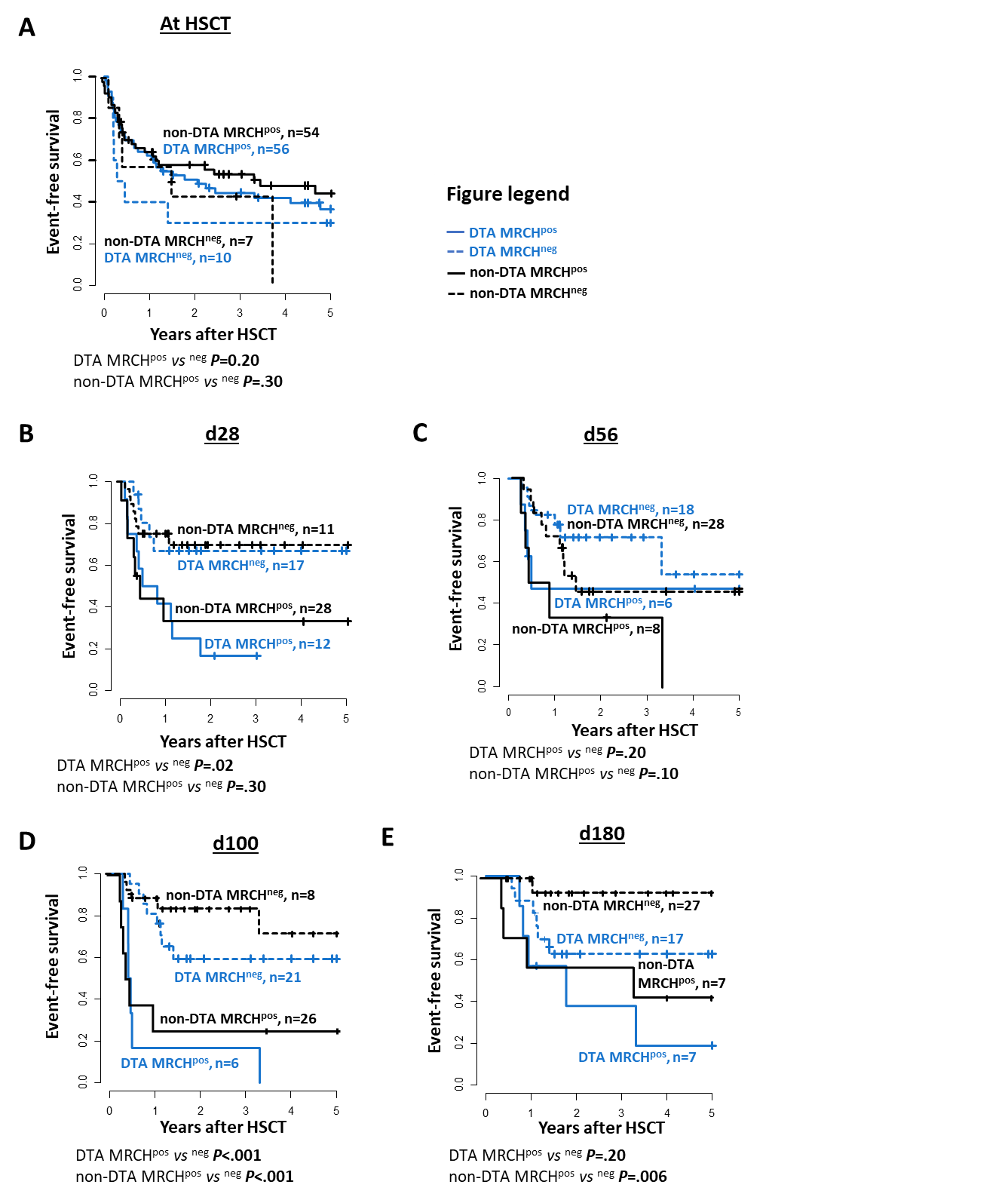
**

**Supplementary Figure S6. Event-free survival of AML patients in CR/CRi undergoing allogeneic HSCT according to the persistence of measurable residual clonal hematopoiesis (MRCH) of DTA or non-DTA mutations at different time points in the first year following HSCT. Outcome according to MRCH detection: (A)** up to 28 days prior tp HSCT **(B)** at day 28 after allogeneic HSCT **(C)** at day 100 after allogeneic HSCT and **(D)** at day 180 after allogeneic HSCT. **(blue straight)** DTA-MRCH^pos^ **(blue dotted)** DTA**-**MRCH^neg^ **(black straight)** non-DTA MRCH^pos^ **(black dotted)** non-DTA MRCH^pos^. **DTA** = mutation in one or more of the genes *DNMT3A, TET2,* and *ASXL1*. **Non-DTA** = mutation in one or more of the genes *SRSF2, JAK2, U2AF1, SF3B1,* and *IDH2* R140Q.

**Supplementary Figure S7.**

**
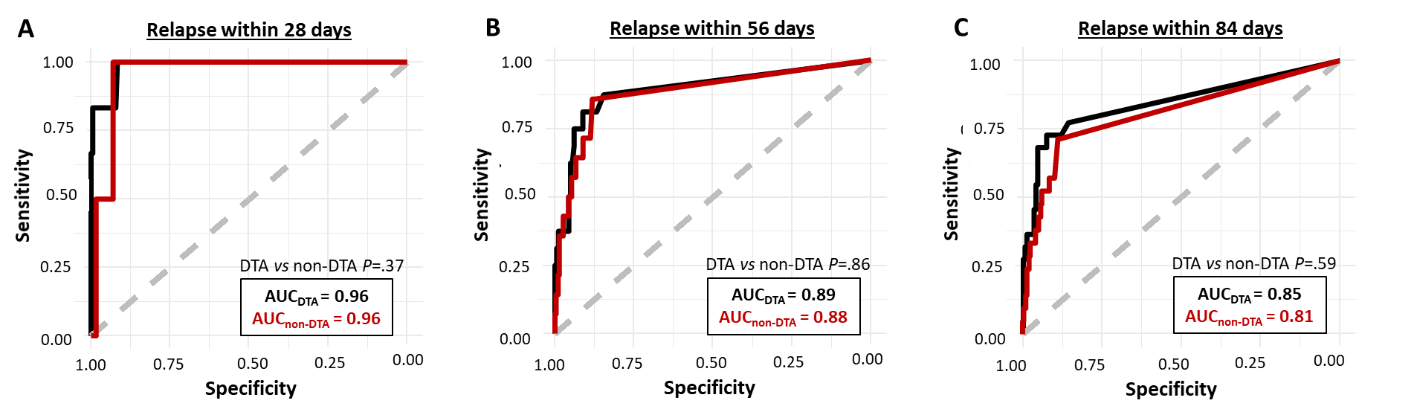
**

**Supplementary Figure S7. ROC curves for relapse prediction in AML patients after allogeneic HSCT according to the MRCH burden depicted separately for DTA (black), and non-DTA mutations (red), (A)** relapse within 28 days after measurement, **(B)** relapse within day 56 days after measurement, and **(C)** relapse within 84 days after measurement. **DTA** = mutation in one or more of the genes *DNMT3A, TET2,* and *ASXL1*. **Non-DTA** = mutation in one or more of the genes *SRSF2, JAK2, U2AF1, SF3B1,* and *IDH2* R140Q.

**Supplementary Figure S8.**

**
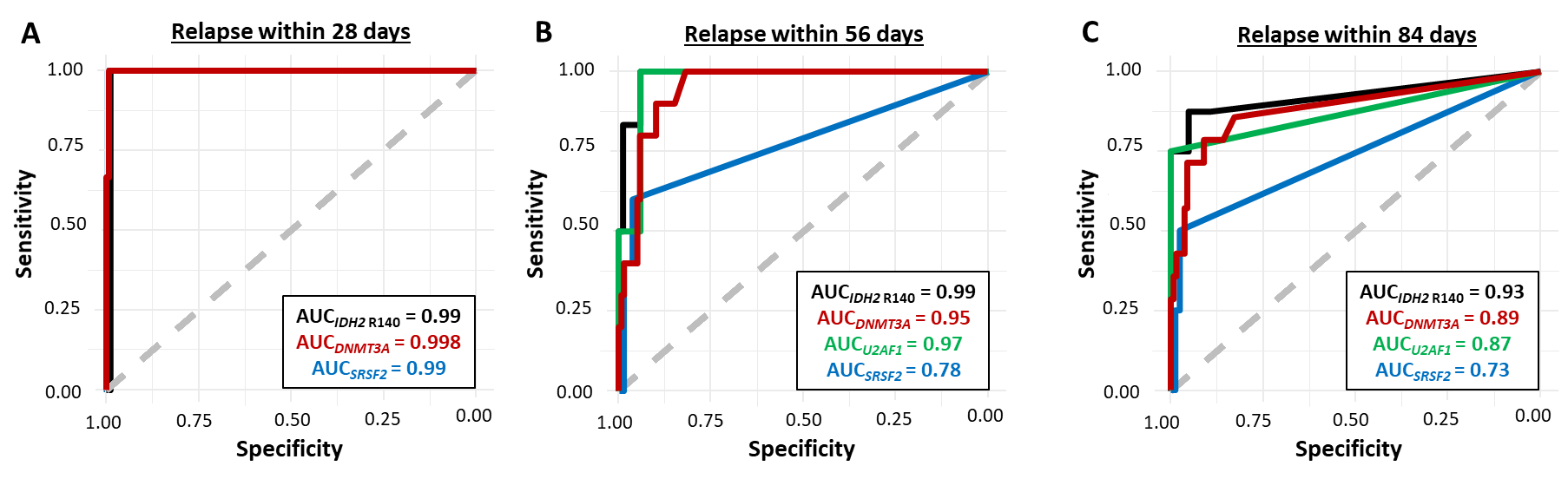
**

**Supplementary Figure S8. ROC curves for relapse prediction in AML patients after allogeneic HSCT according to distinct MRCH mutations, *i.e.* *IDH2* R140 (black), *DNMT3A* (red), *SRSF2* (blue), and *U2AF2* (green). (A)** relapse within 28 days after measurement, **(B)** relapse within day 56 days after measurement, and **(C)** relapse within 84 days after measurement.
